# A Highlight for Non-*Escherichia coli* and Non-*Salmonella* sp. *Enterobacteriaceae* in Dairy Foods Contamination

**DOI:** 10.3389/fmicb.2017.00930

**Published:** 2017-05-24

**Authors:** Angelo M. B. Amorim, Janaína dos Santos Nascimento

**Affiliations:** ^1^Laboratory of Microbiology, Instituto Federal de Educação, Ciência e Tecnologia do Rio de JaneiroRio de Janeiro, Brazil; ^2^Department of Quality Control, Instituto de Tecnologia em Imunobiológicos Bio-Manguinhos, FiocruzRio de Janeiro, Brazil

**Keywords:** enterobacteriaceae, dairy foods, multidrug resistance, virulence factors, biofilm formation, antimicrobial substances, health of consumers, enterobacter

## Background

Dairy products are the base of the diet of many families in all social classes worldwide. However, the high content of milk nutrients and their derivatives, in addition to the near neutral pH and high water activity, provide an ideal environment for the growth of many microorganisms in dairy products (Oladipo and Omo-Adua, 2011). Moreover, during its manufacturing, processing, storage, distribution, and marketing, dairy products may be subject to inadequate hygiene conditions, which can promote spoilage and contamination with pathogenic microorganisms, including *Enterobacteriaceae* (Cusato et al., 2013; Freitas et al., 2013).

Several studies have reported the contamination of dairy products by *Salmonella* sp. and *Escherichia coli*; however, few studies have described the presence of *Enterobacter, Klebsiella*, and *Serratia* species, particularly with regard to the presence of these genera in foods and milk products. In addition, although most studies performed with these bacteria have focused on quantification and identification, some studies have also explored antimicrobial resistance (Samaržija et al., 2012; Zhang et al., 2015) and other virulence factors, such as biofilm (Cherif-Antar et al., 2016), proteolytic enzymes (Chove et al., 2013), and lipolytic enzymes (Masiello et al., 2016).

Cheese is a major dairy product that can be manufactured using nonpasteurized milk; this process is often carried out at farms and small establishments, increasing the potential for contamination by microorganisms, including *Enterobacteriaceae* (Zhang et al., 2015). Even in products subjected to the pasteurization process, the absence of contaminants is not guaranteed because of possible flaws in the process or after pasteurization. In Brazil, the most commonly consumed fresh cheese, “Minas frescal,” has undergone changes in its composition, particularly after campaigns pushing for sodium reduction. However, such changes in composition can exacerbate problems with contamination because sodium is effective in controlling some pathogenic and deteriorative microorganisms, including *Enterobacteriaceae* family members, directly affecting the shelf life of the product and potentially altering its rheological and sensory features (Doyle and Glass, 2010; Cruz et al., 2011; Damaceno et al., 2015).

Contamination in dairy products is not restricted to fresh products. Infant milk formulas and milk powder, which are subjected to various moisture removal processes, have also been reported to be contaminated with representatives of this family. Isolates of *Klebsiella pneumoniae, Citrobacter freundii, Enterobacter cloacae*, and other members of the *E. cloacae* complex have been found in these foods (Oonaka et al., 2010; Sani and Yi, 2011; Yao et al., 2012).

## Resistance to antibiotics

Antibiotics are often used indiscriminately in animals with the goals of prevention and treatment of clinical diseases as well as enhancing growth and development (Fleming et al., 2010; Bosco et al., 2012; Murphy et al., 2016). This routine may be affecting various aspects of the food industry, since antibiotic-resistant microorganisms from animals can be disseminated in different food products (Rolain, 2013).

The use of antibiotics targeting animal growth is still controversial; some researchers argue that consumption of products derived from these animals is not a risk to human health, whereas others have questioned the possibility that bacterial strains with resistance genes may be transmitted by food derived from these animals. For example, Marshall and Levy (2011) reported a study on people working on a farm at which antibiotics were used for animal development; these workers harbored resistant bacteria in their intestinal microbiota, and the same bacterial profile was observed in the animals at the farm.

Notably, such gene transfer can occur, even in the lumen of human and animal intestines, influencing the evolution of newly pathogenic strains (Grotiuz et al., 2006). In addition, milk is considered an excellent culture medium for gene transfer by conjugation and has been reported to have an efficiency that is 10 times higher than that of laboratory culture medium (Verraes et al., 2013).

Some studies have described the occurrence of multidrug resistance (MDR) in members of the *Enterobacteriaceae* family, isolated from dairy products, which do not belong to the species *E. coli* and *S. enterica*, considered classical foodborne pathogens. For example, Chauhan et al. (2013) described the isolation of MDR *K. pneumoniae* from raw milk samples. MDR isolates from the genera *Enterobacter, Citrobacter*, and *Klebsiella*, all of which showed resistance to imipenem, were also described by Fakruddin et al. (2014) in different food samples, including milk powder. More recently, our group has isolated MDR *Enterobacter* spp. from “Minas frescal” cheese and pasteurized milk (Damaceno et al., 2015; Amorim, 2016).

Additionally, studies have shown that *E. cloacae* strains are often isolated from dairy products. However, studies of the virulence factors of these strains and their MDR potential are still scarce.

Among the factors related to acquisition of antimicrobial resistance by representatives of the *Enterobacteriaceae* family, the factor that is most concerning to the scientific community is the ability of these bacteria to produce extended spectrum β-lactamase (ESBL; Thenmozhi et al., 2014; Tekiner and Özpinar, 2016). Inhibitors of ESBL are widely used for the treatment of bacterial infections, particularly for gram-negative bacteria. Therefore, ESBL production can confer resistance to many classes of antibiotics.

The *Enterobacteriaceae* family, which are the greatest producers of ESBL, includes *K. pneumoniae* and *E. coli* strains; these strains have high clinical relevance (Munoz-Price and Weinstein, 2008; Saito et al., 2010) and are unrelated to *Acinetobacter* spp. (*Moraxellaceae* family).

Foods with certain characteristics may facilitate the spread of ESBL bacteria. For example, Calbo et al. (2011) described the transmission of an ESBL*-*producing *K. pneumoniae* strain by food consumption at a health facility; after obtaining negative results for surfaces and professionals in the ward, they determined that the propagation occurred via food, affecting 14% of the food handlers and 35% of hospital kitchen surfaces.

However, the results of various studies have been controversial. Some studies have shown that milk is not a good disseminator of ESBL-producing bacteria, whereas other studies have indicated that milk can facilitate the dissemination of these bacteria. Moreover, most studies showing that ESBL-producing bacteria were present in milk were performed in developing countries, as India, Brazil, and Indonesia, and the results of these studies contrasted with those of studies in developed countries, such as Switzerland (Chauhan et al., 2013; Dahmen et al., 2013; Sudarwanto et al., 2015; Amorim, 2016).

## Other virulence factors

### Biofilm production

Biofilm formation in the dairy industry can occur within a few hours after processing (Mogha et al., 2014). Milk, obviously a major component of dairy products, has characteristics that may promote or present biofilm production on surfaces. Its composition is rich in lipids, proteins, and certain divalent cations, e.g., calcium, which favors the formation of biofilm (Teh et al., 2014; Flint et al., 2015).

Some studies have reported the presence of *Enterobacteriaceae* biofilm producers associated with industrial dairy production plants. Cherif-Antar et al. (2016) found distinct gram-negative bacteria, including *K. pneumoniae, Serratia marcescens*, and *Enterobacter* spp., attached to the stainless steel surfaces of pipes of a milk processing plant.

Notably, these bacteria can be resistant to cleaning products, as described by Malek et al. (2012), who collected samples from farms producing pasteurized milk and skimmed milk powder in Algeria. The entire production line was constantly subjected to sanitization using ammonia- and peracetic acid-based products. Despite this attempt at sanitization, representatives of the *Enterobacter* sp. were still found.

### Proteolytic and lipolytic activity

*Enterobacteriaceae* capable of synthesizing proteolytic and lipolytic enzymes are largely responsible for the deterioration of milk and dairy products, which may cause various issues in the dairy industry (Zajác et al., 2015; Masiello et al., 2016).

In cheese production, for example, these enzymes destabilize casein micelles and may modify or even prevent the coagulation of milk, what can directly affect the formation of the product (Caldera et al., 2015). Another major problem is that these bacteria can cause off-flavor, i.e., can considerably affect the sensory properties of the foods, such as color, odor, flavor, and texture (Böhme et al., 2013; Caldera et al., 2015). Such changes can directly affect the acceptance or rejection of the product by the consumer.

Bacterial synthesis of lipolytic enzymes has also shown to be important for the food industry due the direct influence of these enzymes on sensory properties, particularly flavor and texture. Lipolysis may lead to a process called hydrolytic rancidity, wherein the product develops a sour taste and an unpleasant odor (Carpiné et al., 2010; Krewinkel et al., 2016).

Recently, Masiello et al. (2016) isolated lipolytic representatives of the *Enterobacteriaceae* family (genera *Serratia, Enterobacter*, and *Raoutella*) from pasteurized milk samples. The authors indicated that diverse bacteria found in pasteurized milk, exhibiting phenotypic characteristics such as production of lipolytic and proteolytic enzymes, can result in milk spoilage.

## Production of antimicrobial substances

Some pathogenic bacteria, such as representatives of the *Enterobacteriaceae* family, are capable of producing biologically active compounds known as antimicrobials, acting in a competition niche against its competitors.

These compounds can be purified and used by the food industry as tools to protect against bacteria that cause deterioration in their products, thus increasing their shelf life (Verraes et al., 2013; Damaceno et al., 2015) and maintenance of product characteristics, since they are bactericidal or bacteriostatic without altering the sensory properties of food. These substances have shown to be essential for the food industry since antibiotics cannot be used in foods (Fleming et al., 2010).

Bacteriocins are major antimicrobial substance produced by bacteria; when they are produced by commensal bacteria in the intestinal tract of animals, they may have an important role in elimination of MDR microorganisms, without major changes in intestinal flora (Kommineni et al., 2015), since they are degraded by enzymes of the digestive system and have probiotic properties (Rosa et al., 2016).

In a previous work carried out by our research group, two representatives of *Enterobacter* sp. and nine other representatives of the *Enterobacteriaceae* family were found to produce antimicrobial substances against strains *E. coli* and *S. enterica* used as indicators (Damaceno et al., 2015). These two species of bacteria are among the major causes of foodborne illnesses, and this previous work suggested that classical *Enterobacteriaceae* pathogens can be inhibited by other representatives of the same family, which could justify their absence or low levels in some dairy foods.

## Conclusions

Dairy products are potential vehicles for microorganisms from the *Enterobacteriaceae* family, which can exhibit MDR to available antimicrobials, reduced susceptibility phenotypes to carbepenens (KPC), and ESBL production and produce biofilm, proteolytic enzymes, lipolytic enzymes, and antimicrobial substances, providing advantages for the bacteria in a competitive niche. All these factors represent potential risks to the health of consumers of dairy products, particularly immunocompromised consumers.

In the supply chain of dairy products, including all stages (e.g., production lines, transport, and storage), good manufacturing practices and hygiene, as well as best practices in commercialization, must be followed mainly for products consumed without any prior processing. Additionally, the absence of classic pathogens, such as *Salmonella* sp. and *E. coli*, does not indicate that the product is fit for consumption, since other potentially pathogenic bacteria of the same family may be present in the food. Thus, testing for *Enterobacteriaceae*, including species that are not yet assessed according to regulator standards, may offer a better view of the quality, sanitary conditions, and safety of dairy foods.

## Author contributions

AA and JN wrote this article.

### Conflict of interest statement

The authors declare that the research was conducted in the absence of any commercial or financial relationships that could be construed as a potential conflict of interest.

## References

[B1] Amorim A. M. B. (2016). Avaliação da Multirresistência a Drogas e da Produção de Outros Fatores de Virulência por Isolados de Enterobacteriaceae Provenientes de Produtos Lácteos. Master's thesis, Instituto Federal de Educação, Ciência e Tecnologia do Rio de Janeiro, Rio de Janeiro.

[B2] Böhme K. Fernández-No I. C. Barros-Velázquez J. Gallardo J. M. Calo-Mata P. Cañas B. (2013). Species identification of food spoilage and pathogenic bacteria by MALDI-TOF mass fingerprinting. J. Prot. Res. 9, 3169–3183. 10.1021/pr100047q 20408567

[B3] Bosco K. J. Kaddu-Mulindwa D. H. Asiimwe B. B. (2012). Antimicrobial drug resistance and plasmid profiles of Salmonella isolates from humans and foods of animal origin in Uganda. Adv. Infect. Dis. 2, 151–155. 10.4236/aid.2012.24025

[B4] Calbo E. Freixas N. Xercavins M. Riera M. Nicolás C. Monistrol O.. (2011). Foodborne nosocomial outbreak of SHV1 and CTX-M-15–producing *Klebsiella pneumoniae*: epidemiology and control. Clin. Infect. Dis. 52, 743–749. 10.1093/cid/ciq238 21367727

[B5] Caldera L. Arioli S. Stuknyte M. Scarpellini M. Franzetti L. (2015). Setup of a rapid method to distinguish among dead, alive, and viable but not cultivable cells of *Pseudomonas* spp. in mozzarella cheese. J. Dairy Sci. 98, 8368–8374. 10.3168/jds.2015-9677 26433412

[B6] Carpiné D. Dagostin J. L. A. Santa H. S. D. Alvarez D. C. Terra N. N. Santa O. R. D. (2010). Atividade proteolítica e lipolítica de bactérias lácticas isoladas de salames artesanais. Ambiência 6, 125–132.

[B7] Chauhan S. Farooq U. Singh V. Kumar A. (2013). Determination of prevalence and antibacterial activity of ESBL (Extended Spectrum Beta-lactamases) producing *Klebsiella* species isolated from raw milk of Doon Valley in India. Int. J. Pharm. Biol. Sci. 4, 417–423.

[B8] Cherif-Antar A. Moussa-Boudjemâa B. Didouh S. Medjahdi K. Mayo B. Flórez A. B. (2016). Diversity and biofilm-forming capability of bacteria recovered from stainless steel pipes of a milk-processing dairy plant. Dairy Sci. Technol. 96, 27–38. 10.1007/s13594-015-0235-4

[B9] Chove L. M. Issa-Zacharia A. Grandison A. S. Lewis M. J. (2013). Proteolysis of milk heated at high temperatures by native enzymes analysed by trinitrobenzene sulphonic acid (TNBS) method. Afr. J. Food Sci. 7, 232–237. 10.5897/AJFS13.0987

[B10] Cruz A. G. Faria J. A. F. Pollonio M. A. R. Bolini H. M. A. Celeghini R. M. S. Granato D. (2011). Cheeses with reduced sodium content: effects on functionality, public health benefits and sensory properties. Trends Food Sci. Tech. 22, 276–291. 10.1016/j.tifs.2011.02.003

[B11] Cusato S. Gameiro A. H. Corassin C. H. Sant'ana A. S. Cruz A. G. Faria J. A.. (2013). Food safety systems in a small dairy factory: implementation, major challenges, and assessment of systems performances. Foodborne Pathog. Dis. 10, 6–12. 10.1089/fpd.2012.1286 23153286

[B12] Dahmen S. Métayer V. Gay E. Madec J.-Y. Haenni M. (2013). Characterization of extended-spectrum beta-lactamase (ESBL)-carrying plasmids and clones of Enterobacteriaceae causing cattle mastitis in France. Vet. Microbiol. 162, 793–799. 10.1016/j.vetmic.2012.10.015 23127568

[B13] Damaceno H. F. B. Freitas Junior C. V. Marinho I. L. Cupertino T. R. Costa L. E. O. Nascimento J. S. (2015). Antibiotic resistance versus antimicrobial substances production by gram-negative foodborne pathogens isolated from minas frescal cheese: heads or tails? Foodborne Pathog. Dis. 12, 297–301. 10.1089/fpd.2014.1876 25622265

[B14] Doyle M. E. Glass K. A. (2010). Sodium reduction and its effect on food safety, food quality, and human health. Comp. Rev. Food Sci. Food Safety 9, 44–56. 10.1111/j.1541-4337.2009.00096.x 33467812

[B15] Fakruddin Md. Rahaman M. Ahmed M. M. Hoque M. (2014). Antimicrobial resistance and virulence factors of Enterobacteriaceae isolated from food samples of Bangladesh. Int. J. Microbiol. Immunol. Res. 3, 12–18.

[B16] Fleming L. R. Bolzan D. N. Nascimento J. S. (2010). Antimicrobial substances produced by coliform strains active against foodborne pathogens. Foodborne Pathog. Dis. 7, 243–247. 10.1089/fpd.2009.0333 19895262

[B17] Flint S. Jamaludin N. M. Somerton B. Palmer J. Brooks J. (2015). The effect of milk composition on the development of biofilms, in Biofilms in the Dairy Industry, eds Teh K. H. Flint S. Brooks J. Knight G. (Chichester: John Wiley & Sons), 36–48. 10.1002/9781118876282.ch3

[B18] Freitas R. Brito M. A. Nero L. A. De Carvalho A. F. (2013). Microbiological safety of Minas Frescal Cheese (MFC) and tracking the contamination of *Escherichia coli* and *Staphylococcus aureus* in MFC processing. Foodborne Pathog. Dis. 10, 951–955. 10.1089/fpd.2013.1525 23909773

[B19] Grotiuz G. Sirok A. Gadea P. Varela G. Schelotto F. (2006). Shiga toxin 2-producing Acinetobacter haemolyticus associated with a case of bloody diarrhea. J. Clin. Microbiol. 44, 3838–3841. 10.1128/JCM.00407-06 17021124 PMC1594762

[B20] Kommineni S. Bretl D. J. Lam V. Chakraborty R. Hayward M. Simpson P.. (2015). Bacteriocin production augments niche competition by enterococci in the mammalian gastrointestinal tract. Nature 526, 719–722. 10.1038/nature15524 26479034 PMC4978352

[B21] Krewinkel M. Baur C. Kranz B. Von Neubeck M. Wenning M. Scherer S. (2016). A sensitive and robust method for direct determination of lipolytic activity in natural milk environment. Food Anal. Method 9, 646–655. 10.1007/s12161-015-0233-4

[B22] Malek F. Moussa-Boudjemâa B. Khaouani-Yousfi F. Kalai A. Kihel M. (2012). Microflora of biofilm on Algerian dairy processing lines: an approach to improve microbial quality of pasteurized milk. Afr. J. Microbiol. Res. 6, 3836–3844. 10.5897/AJMR11.1120

[B23] Marshall B. M. Levy S. B. (2011). Food animals and antimicrobials: impacts on human health. Clin. Microbiol. Rev. 24, 718–733. 10.1128/CMR.00002-11 21976606 PMC3194830

[B24] Masiello S. N. Martin N. H. Trmčić A. Wiedmann M. Boor K. J. (2016). Identification and characterization of psychrotolerant coliform bacteria isolated from pasteurized fluid milk. J. Dairy Sci. 99, 130–140. 10.3168/jds.2015-9728 26547640

[B25] Mogha K. V. Shah N. P. Prajapati J. B. Chaudhari A. R. (2014). Biofilm - A threat to dairy industry. Indian J. Dairy Sci. 67:6.

[B26] Munoz-Price L. S. Weinstein R. A. (2008). Acinetobacter infection. N.Engl. J. Med. 358, 1271–1281. 10.1056/NEJMra070741 18354105

[B27] Murphy C. P. Fajt V. R. Scott H. M. Foster M. J. Wickwire P. McEwen S. A. (2016). Scoping review to identify potential non-antimicrobial interventions to mitigate antimicrobial resistance in commensal enteric bacteria in North American cattle production systems. Epidemiol. Infect. 144, 1–18. 10.1017/S0950268815000722 25904121 PMC4697299

[B28] Oladipo I. C. Omo-Adua R. O. (2011). Antibiotics resistance among bacteria isolated from evaporated milk. Asian J. Biol. Sci. 4, 77–83. 10.3923/ajbs.2011.77.83

[B29] Oonaka K. Furuhata K. Hara M. Fukuyama M. (2010). Powder infant formula milk contaminated with *Enterobacter sakazakii*. Jpn. J. Infect. Dis. 63, 103–107. 20332571

[B30] Rolain J. (2013). Food and human gut as reservoirs of transferable antibiotic resistance encoding genes. Front. Microbiol. 4:173. 10.3389/fmicb.2013.00173 23805136 PMC3690338

[B31] Rosa L. J. B. Esper L. M. R. Cabral J. P. L. G. Franco R. M. Cortez M. A. S. (2016). Viability of probiotic micro-organism *Lactobacillus acidophilus* in dairy chocolate dessert and its action against foodborne pathogens. Ciênc. Rural 46, 368–374. 10.1590/0103-8478cr20141864

[B32] Saito R. Koyano S. Nagai R. Okamura N. Koike K. (2010). Evaluation of a Chromogenic medium for extended-spectrum beta-lactamase-producing Enterobacteriaceae. Lett. Appl. Microbiol. 51, 704–706. 10.1111/j.1472-765X.2010.02945.x 21117288

[B33] Samaržija D. Zamberlin S. Pogačić T. (2012). Psychrotrophic bacteria and their negative effects on milk and dairy products quality. Mljekarstvo 62, 77–95.

[B34] Sani N. A. Yi L. Y. (2011). Enterobacteriaceae, Cronobacter (Enterobacter) sakazakii and microbial population in infant formula products in the Malaysian market. Sains Malays. 40, 345–351.

[B35] Sudarwanto M. Akinedem O. Odenthal S. Gross M. Usleber E. (2015). Extended-spectrum β-lactamase (ESBL)–producing *Klebsiella pneumoniae* in bulk tank milk from dairy farms in Indonesia. Foodborne Pathog. Dis. 12, 585–590. 10.1089/fpd.2014.1895 26135892

[B36] Teh K. H. Flint S. Palmer J. Andrewes P. Bremer P. Lindsay D. (2014). Biofilm − An unrecognised source of spoilage enzymes in dairy products? Int. Dairy J. 34, 32–40. 10.1016/j.idairyj.2013.07.002

[B37] Tekiner I. H. Özpinar H. (2016). Occurrence and characteristics of extended spectrum beta-lactamases-producing Enterobacteriaceae from foods of animal origin. *Bra*z. J. Microbiol. 47, 444–451. 10.1016/j.bjm.2015.11.034 PMC4874675 26991276

[B38] Thenmozhi S. Moorthy K. Sureshkumar B. T. Suresh M. (2014). Antibiotic resistance mechanism of ESBL producing Enterobacteriaceae in clinical field: a review. Int. J. Pure App. Biosci. 2, 207–226.

[B39] Verraes C. Van Boxstael S. Van Meervenne E. Van Coillie E. Butaye P. Catry B.. (2013). Antimicrobial resistance in the food chain: a review. Int. J. Environ. Res. Public Health 10, 2643–2669. 10.3390/ijerph10072643 23812024 PMC3734448

[B40] Yao K. Zinzendorf N. Y. Bohoua G. Kouassi K. A. Koua A. Kouadio L. K. (2012). Occurrence of *Enterobacter sakazakii* (Cronobacter) and Other Enterobacteriaceae in commercial powdered infant formula in Abidjan, Ivory Coast. Food Nutr. Sci. 3, 822–226. 10.4236/fns.2012.36110

[B41] Zajác P. Čapla J. Vietoris V. Zubrická S. Čurlej J. (2015). Effects of storage on the major constituents of raw milk. Potravinarstvo 9, 375–381. 10.5219/518

[B42] Zhang R. Huo W. Zhu W. Mao S. (2015). Characterization of bacterial community of raw milk from dairy cows during subacute ruminal acidosis challenge by high-throughput sequencing. J. Sci. Food Agric. 95, 1072–1079. 10.1002/jsfa.6800 24961605

